# Supporting middle-cadre health care workers in Malawi: lessons learned during implementation of the PALM PLUS package

**DOI:** 10.1186/1472-6963-14-S1-S8

**Published:** 2014-05-12

**Authors:** Sumeet Sodhi, Hastings Banda, Damson Kathyola, Martias Joshua, Faye Richardson, Emmay Mah, Hayley MacGregor, Emmanuel Kanike, Sandy Thompson, Lara Fairall, Eric Bateman, Merrick Zwarenstein, Michael J Schull

**Affiliations:** 1 Dignitas International, 20 Toronto Street, Suite 1220, Toronto Ontario, M5C 2B8, Canada; 2 Department of Family and Community Medicine, Faculty of Medicine, University of Toronto, 500 University Avenue, Toronto, Ontario, M5G 1V7, Canada; 3 Department of Family and Community Medicine, University Health Network, Toronto Western Hospital, 399 Bathurst Street, Toronto, Ontario M5T 2S8, Canada; 4 Research for Equity and Community Health (REACH) Trust, P0B 1597, Lilongwe, Malawi; 5 Ministry of Health, Malawi, POB 3, Lilongwe, Malawi; 6 Zomba Central Hospital, Kamuzu Highway, Zomba, Malawi; 7 Institute of Development Studies, University of Sussex, Library Road, Brighton, BN1 9RE, UK; 8 Knowledge Translation Unit, University of Cape Town Lung Institute, University of Cape Town, PO Box 34560, Groote Schuur 7937, South Africa; 9 Sunnybrook Health Sciences Centre, 2075 Bayview Ave, Toronto, Ontario, M4N 2M5, Canada; 10 Department of Medicine, University of Toronto, 200 Elizabeth Street, Toronto, Ontario M5G 2C4, Canada; 11 Department of Health Policy, Management and Evaluation, 155 College Street, Suite 425, Toronto, Ontario M5T 3M6, Canada

**Keywords:** training, educational outreach, task-shifting, continuing professional development, antiretroviral treatment, integrated primary care, nurses, clinical officers, medical assistants, health surveillance assistants, guideline, train-the-trainer, south-south collaboration, formation, sensibilisation, délégation de tâches, formation continue, soins primaires intégrés, personnel infirmier, personnel clinique, auxiliaires médicaux, adjoints à la surveillance sanitaire, lignes directrices, formation des formateurs, collaboration Sud-Sud

## Abstract

**Background:**

The government of Malawi is committed to the broad rollout of antiretroviral treatment in Malawi in the public health sector; however one of the primary challenges has been the shortage of trained health care workers. The Practical Approach to Lung Health Plus HIV/AIDS in Malawi (PALM PLUS) package is an innovative guideline and training intervention that supports primary care middle-cadre health care workers to provide front-line integrated primary care. The purpose of this paper is to describe the lessons learned in implementing the PALM PLUS package.

**Methods:**

A clinical tool, based on algorithm- and symptom-based guidelines was adapted to the Malawian context. An accompanying training program based on educational outreach principles was developed and a cascade training approach was used for implementation of the PALM PLUS package in 30 health centres, targeting clinical officers, medical assistants, and nurses. Lessons learned were identified during program implementation through engagement with collaborating partners and program participants and review of program evaluation findings.

**Results:**

Key lessons learned for successful program implementation of the PALM PLUS package include the importance of building networks for peer-based support, ensuring adequate training capacity, making linkages with continuing professional development accreditation and providing modest in-service training budgets. The main limiting factors to implementation were turnover of staff and desire for financial training allowances.

**Conclusions:**

The PALM PLUS approach is a potential model for supporting mid-level health care workers to provide front-line integrated primary care in low and middle income countries, and may be useful for future task-shifting initiatives.

## Abstract (fr)

**Résumé:**

**Contexte**

Le gouvernement du Malawi souhaite déployer le traitement antirétroviral dans le secteur de la santé publique à l’échelle du pays. Toutefois, l’un des principaux obstacles auxquels il se heurte est la pénurie de travailleurs de la santé adéquatement formés. L’outil d’approche pratique de la santé pulmonaire et du VIH/sida au Malawi (PALM PLUS) comporte une initiative de formation et d’orientation qui soutient les travailleurs intermédiaires de la santé dans la prestation de soins de première ligne intégrés. Le présent article décrit les enseignements tirés du déploiement de l’outil PALM PLUS.

**Méthodes et conception**

Un outil clinique fondé sur un algorithme et des lignes directrices axées sur les symptômes ont été adaptés en fonction du contexte malawite. Un programme de formation complémentaire fondé sur les principes de la sensibilisation a été mis sur pied. De plus, on a mis en oeuvre tout un éventail d’approches de formation pour déployer l’outil PALM PLUS dans 30 centres de santé, à l’intention du personnel clinique, des auxiliaires médicaux et du personnel infirmier. On s’est employé à dégager des enseignements tout au long du déploiement de l’outil, au fil des interactions avec les partenaires et les participants au programme de formation ainsi que pendant l’examen des constatations ayant émané de l’évaluation du programme.

**Résultats**

Les principaux enseignements tirés du déploiement de l’outil PALM PLUS ont trait à l’importance de mettre en place des réseaux de soutien par les pairs, de s’assurer que les capacités de formation sont adéquates, de tisser des liens avec des organes de certification aux fins de la formation continue et de débloquer de modestes budgets pour la formation en milieu de travail. Les principaux facteurs qui entravent le déploiement sont le roulement du personnel et le manque de fonds alloués à la formation.

L’outil PALM PLUS pourrait constituer un modèle à suivre pour soutenir les travailleurs intermédiaires de la santé dans la prestation de soins de première ligne intégrés dans les pays à faible revenu et les pays à revenu intermédiaire. Il pourrait aussi s’avérer utile dans les initiatives de délégation de tâches.

## Background

Globally, there were an estimated 34 million people living with HIV in 2011, with over eight million on antiretroviral treatment (ART); in Malawi, around 900,000 people were living with HIV/AIDS, with over 300,000 on ART [1]. Since 2004, the government of Malawi has committed to the broad rollout of ART services in the public health sector to facilitate increased access to treatment, care and support for people living with HIV/AIDS through decentralization of HIV/AIDS services to rural primary care facilities [2]. One of the primary challenges of rolling out ART services while maintaining quality of care has been the shortage of trained health care workers (HCWs). The majority of patient care in Malawi, including ART, is delivered by middle-cadre HCWs (clinical officers, medical assistants, and nurses) at peri-urban and rural health centres (HCs), where over 50% of health care posts are vacant [3]. Therefore, innovative approaches to attract, support, and retain HCWs are required to make the most efficient use of existing human resources. In addition to the lack of trained HCWs, decentralization of ART to HCs has highlighted the need to integrate HIV services with primary care, which also requires innovative approaches to support HCWs and health systems.

Traditionally, training for HCWs at HCs in Malawi has been disease and guideline specific and conducted at a central location away from the HC through didactic sessions led by outside expert trainers. The Practical Approach to Lung Health Plus HIV/AIDS in Malawi (PALM PLUS) package is a novel guideline and training intervention that targets primary care middle-cadre HCWs with the objective of supporting provision of front-line integrated primary care.

The PALM PLUS package involved development of a set of algorithm- and symptom-based guidelines and training program to facilitate utilization of guidelines in the workplace. The PALM PLUS guidelines are evidence-based and tailored to the existing Malawi model of care, and take the form of a spiral-bound booklet referred to as a clinical tool or ‘job-aid’. The clinical tool provides integrated clinical advice for the following priority primary care conditions: HIV/AIDS (including prevention of mother to child transmission), tuberculosis, malaria, asthma, chronic obstructive pulmonary disease, and sexually transmitted infections. Although the clinical tool was designed to be simple and self-explanatory, the PALM PLUS package included a training program on how to use the guideline, since evidence has shown that the impact of passive guideline dissemination on practice is minimal [4]. The training program was based on the knowledge translation principles of educational outreach, an approach known to be effective in changing HCW behavior and practice [5]. These principles included the provision of short-face-to-face, small group (10-15 participants), in-service and on-site sessions using case-based training facilitated by a trusted peer, which allowed HCWs to discover proper diagnosis and management by navigating through the guideline. Table 1 shows the key differences between the PALM PLUS training approach compared to the standard approach in Malawi. The PALM PLUS package was adapted from a similar intervention from South Africa called Practical Approach to Lung Health in South Africa (PALSA), which has been shown to have achieved clinically important and statistically significant improvements in quality of care and health outcomes [6].

**Table 1:** Comparison of standard and PALM PLUS training approaches of health care workers

Standard training approach by Malawi Ministry of Health	PALM PLUS training approach
Centralized by health district	Decentralized (on-site at individual clinics)

Presentations (primarily didactic and unidirectional) by external experts	Educational outreach (facilitative, case-based) by trained peer facilitators

Large groups (50-100 participants); little discussion or interaction between participants and trainer	Small groups (10-15 participants); discussion and interaction among participants and between participants and trainer encouraged

Disease specific care	Symptom-based and integrated care

Written, narrative guidelines with figures, tables and algorithms, text-heavy	PALM PLUS clinical tool consisting of algorithm- and symptom-based guidelines in a single spiral-bound book, with graphical interface

Financial training allowances given to individual participants	No financial training allowances given to individual participants

Individual staff trained one at a time	All staff at a health facility learn at the same time

Conducted over 1–14 consecutive days	Conducted over 8–12 training sessions over a three-to-four month period

The process of adapting PALSA to PALM involved extensive guideline revisions based on Malawi national guidelines, customization of training materials, and adjustments to the selection process for trainers, whereby PALM PLUS trainers were recruited from all the different mid-level HCW cadres (clinical officer, medical assistant, nurse) as opposed to exclusive utilization of nurse-managers as trainers for PALSA [7]. The PALM PLUS intervention was implemented with full collaboration with the Malawi Ministry of Health (MoH), the Nurses and Midwives Council of Malawi, the Medical Council of Malawi and the Zomba District Health Office. The main implementation partner was Dignitas International, a Canadian-based non-governmental organization working in partnership with the MoH since 2004 to scale up HIV services in Zomba District, and the main evaluation partner was REACH Trust, a Malawian multi-disciplinary health research organization.

## Methods

### Training program

**Figure 1:**
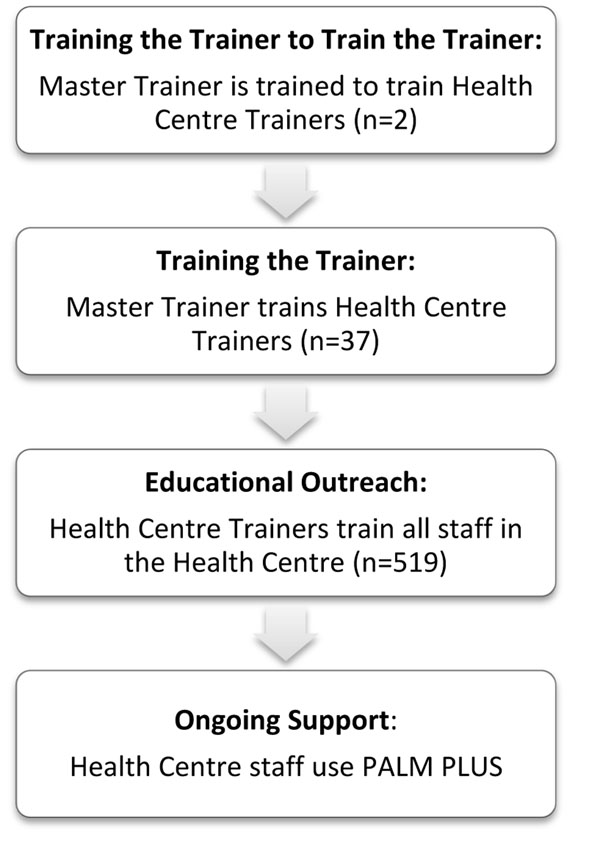
PALM PLUS Cascade Training Model

Once the clinical tool was adapted and approved by all stakeholders, a training program was established using a cascade training approach (Figure 1). First, in 2009, a master trainer from Malawi was trained and certified in South Africa by experienced trainers from the PALSA program at the Knowledge Translation Unit at the University of Cape Town (KTU) over a 10-day period, in a program called “Training the Trainer to Train the Trainer”. The new master trainer then trained 13 HC trainers in January 2010 over a five-day period in Malawi, with on-site support from visiting trainers from the KTU, bringing the total of PALM PLUS HC trainers to 14, one for each intervention site (the master trainer also functioned as a HC trainer). HC trainers were selected in collaboration with the Zomba District Health Office. This five-day training for HC trainers was referred to as “Training the Trainer to Train (TtTtT)”. The master trainer provided support for the HC trainers through close face-to-face follow up in the first six weeks after the TtTtT. Additional support was also offered to the HC trainers throughout the PALM PLUS intervention by way of quarterly meetings, newsletters, recognition programs, and communication though web-based SMS, e-mail, and telephone.

Each HC trainer was responsible for conducting educational outreach training sessions for the PALM PLUS package among all the middle-cadre HCWs at their assigned HC, and was often referred to as the “resident trainer”. The educational outreach training sessions were short (1–1.5 hours), face-to-face in small groups, in-service and on-site (before or after clinic or on weekends), and consisted of 8–12 training sessions over a three-to-four month period. HC trainers were available to HCWs to advise on use of the PALM PLUS clinical tool and to provide mentorship for integrating the use of the clinical tool into daily clinical practice. All PALM PLUS participants who completed a minimum of six educational outreach sessions were issued a certificate and awarded continuing professional development (CPD) credits with their profession’s licensing authority. HC trainers were similarly awarded CPD credits. No financial incentives were offered either to individual PALM PLUS participants or HC trainers.

### Program evaluation

The PALM PLUS package is being evaluated through a mixed methods approach, which includes programmatic evaluation as well as a population-based un-blinded stratified cluster-randomized trial, with the HC as the unit of randomization. Primary outcomes of interest for the trial are HCW satisfaction and retention at the individual HCW level, and clinical outcomes at the HC level, and both quantitative and qualitative methods are being utilized for data collection and analysis. Detailed descriptions of both the adaptation process from PALSA and the trial protocol have been previously published [7,8]. This paper will focus on programmatic lessons learned during the implementation of the PALM PLUS package.

In December 2010, thirty HCs in Zomba District were chosen to participate in the PALM PLUS intervention in collaboration with the Zomba District Health Office. As the intervention was being evaluated through a cluster-randomized trial, 14 intervention sites and 16 control sites were randomly allocated to either receive the PALM PLUS package or continue with standard training opportunities. Random allocation was stratified by funding source (government-funded HC vs. other-funded HC) and HC size (defined by the number of HCWs at that centre). Plans were made to roll out the PALM PLUS package to the control sites at the end of the study trial period, as well as other health facilities not included in the study. Ongoing routine training using the standard MoH approach continued at both the intervention and control sites.

In preparation for the roll out of the PALM PLUS package to control sites and Zomba Central Hospital (excluded from the cluster-randomized trial due to its tertiary care level) at the end of the study trial period, and in response to unexpectedly high turnover of staff (of both the master trainer and HC trainers), two TtTtTs, program evaluation activities, and revisions of the clinical tool were conducted from July to December 2011. The additional TtTtTs were done in the same manner as the first TtTtT, adding an additional 24 new HC trainers to the pool. The revisions of the clinical tool were done primarily to reflect the new integrated ART guidelines released by the MoH in 2011.

Program evaluation activities included routine end-of-training surveys, quarterly program reports, and a formal end-of-program evaluation. For the program evaluation, ten control sites were selected where no PALM PLUS training or guideline distribution had occurred at the time of data collection; and eleven intervention sites were selected where training in PALM PLUS had occurred. An effort was made to ensure a spread across government-funded and non-government-funded HC as well as urban and rural HC. With respect to the intervention sites, only staff that had a received a minimum of four sessions of PALM PLUS training were included. Data collection comprised of face-to-face structured interviews and focus group discussions. Thematic analysis of the data was done by triangulating the information gathered through the different modalities (training surveys, quarterly reports, program evaluation interviews, and focus group discussions).

Interviews were conducted with HCW, namely medical assistants, clinical officers, and nurses (including nurse midwives). The questions focused on training, clinical confidence, factors which affected and guided clinical practices. Additional questions were posed to clinical staff in the intervention sites regarding the training and application of PALM PLUS. The questions specific to PALM PLUS were placed right at the end of the interview schedule, in an attempt to minimize bias towards PALM PLUS in the responses given. Interviews were recorded and transcribed in instances where consent was given to do so; if consent to record was withheld, the responses were written down on paper.

Focus group discussions were conducted at all ten of the control and nine out of eleven intervention HCs with non-clinical cadres of staff, namely laboratory assistants, health surveillance assistants, and pharmacy assistants. The aim was to assess the extent of their involvement in training and their expressed needs in this regard.

Individual participant consent was obtained for evaluation activities and the research protocol, including both quantitative and qualitative methodologies, approved by the National Health Research Sciences Council in Malawi (Protocol #687). The results of the program evaluation were utilized for quality improvement and refinement of the PALM PLUS package, as well as for donor reporting.

## Results

From January 2010 to December 2011, a total of 37 HC trainers were trained during three TtTtTs (13 in January 2010, 12 in July 2011, and 12 in October 2011): 27 were from primary HCs, five from hospitals (public, army, police, Christian Health Association of Malawi), two from the Zomba District Health Office and three from Dignitas International. By December 2011, 30 HC trainers (81%) were active and had conducted PALM PLUS educational outreach sessions in the last six months, four had moved and/or left their posts, two were on academic leave and one was unknown; all seven of the inactive HC trainers were from those trained in January 2010.

During the same time period, 386 HCWs in the target group (clinical officers, medical assistants, and nurses) were trained in utilization of the PALM PLUS package; 168 (44%) completed the minimum required six sessions. 292 PALM PLUS educational outreach sessions occurred in this time period. An additional 133 health centre staff not in the target group joined the educational outreach sessions, but only 27 (20%) completed the minimum required six sessions. The additional health centre staff included health surveillance assistants, laboratory technicians, pharmacists, X-ray technicians, and occupational/physical therapists.

For the program evaluation, a total of 40 interviews were conducted in the control sites and a total of 18 interviews in the intervention sites. Even though over 40 staff were assessed as eligible for interview in the intervention sites, there were many constraints in the data collection that meant that only 18 interviews were obtained. Most significantly, it was hard to locate enough staff who had received 4 PALM PLUS sessions primarily due to staff turnover at the HCs. An even spread of respondents was obtained across the three clinical staff cadres in both control and intervention sites.

Key lessons learned were identified by the implementation partners through review of program documentation and evaluation findings. The lessons learned can be classified into three categories: supporting HCWs, supporting trainers and mentors, and supporting health systems. Table 2 provides an overview of the lessons learned.

**Table 2:** Lessons learned during implementation of the PALM PLUS package

Health Care Worker Support
• Regular, sustained peer-based support is an essential component for an in-service training program • Educational outreach can assist with training of new staff in the context of frequent staff turnover as it may facilitate peer-based support networks • Educational outreach training methods and symptom-based algorithmic guidelines may be useful for training multidisciplinary staff if adapted to their scope of practice • Financial incentives are highly valued by health care workers for motivation, but this should not inhibit the development of alternative incentive strategies if expectations of success consider the broader health sector compensation challenges in resource-limited settings

**Trainer and Mentor Support**

• Peer-based support and mentorship is equally essential for trainers and mentors • Building sufficient training capacity is critical, and risk mitigation strategies for potential staff turnover should be considered early in the planning stages of implementation • Sustained engagement of motivated trainers is key to training program success; this can be achieved through establishing both formal and informal networks for information sharing

**Health System Support**

• Linking a training program to continuing professional development credits motivates health care workers and trainers and mentors in uptake of training programs • Having a small budget available for in-service training at health facility also increases uptake of in-service training programs – a little goes a long way! • Regular maintenance of training logs helps with efficient and equitable allocation of training to health care workers, and an on-line, web-based tool is a convenient way to implement this

### Health care worker support

Peer-based support, an essential component of educational outreach, was identified by PALM PLUS participants as a key source of support and motivation. All HCWs, both at control and intervention sites, valued the ability to draw on the experience of other colleagues for advice if they were unsure about the management of a patient, including being able to draw on formal and informal networks, and access support by phone. Among PALM PLUS participants, access to the HC trainer was viewed as an advantage for this type of support, and the HC trainer was considered to have a mentorship role.

A major disincentive among PALM PLUS participants to sustain engagement in the training program was the lack of financial training allowances. Training allowances to cover transportation, meals, and accommodation are often provided to HCWs when travelling off-site to attend trainings. Educational outreach sessions were conducted on-site and in-service, thus no training allowances were provided for PALM PLUS participants, which could have contributed to the low completion rate of educational outreach sessions. Any future implementation of the PALM PLUS package should consider continuing the utilization of alternate strategies for motivating HCWs to complete training, such as recognition programs and granting CPD credit, as well as examining HCW motivation in the context of the broader health sector compensation challenges in resource-limited settings such as Malawi.

Transfer of staff between HCs was another challenge and also contributed to the low completion rate of training sessions among PALM PLUS participants in the target group, since new staff at the HC would start their educational outreach sessions midway through the implementation. In a low-resource context such as Zomba District, where health posts are often vacant, HCWs are regularly redistributed among health facilities by the District Health Office (DHO) in an attempt to optimize deployment of the limited human resources and through a request-for-transfer system. While this redistribution made the implementation process more difficult, the opportunity provided by educational outreach sessions for ‘old’ staff to interact and mentor ‘new’ staff could be utilized as a potential strategy to facilitate peer-based support in future initiatives.

Some HC staff outside the target group, primarily health surveillance assistants and laboratory technicians, joined the PALM PLUS educational outreach sessions on their own accord, and accounted for just over one-quarter (133 out of 519) of participants who participated in at least one educational outreach session. However, the non-target group staff had low completion rates, and found that either the content of the PALM PLUS package was not fully relevant or applicable to their scope of practice or at their level of training. Health surveillance assistants in particular reported that they would be eager to learn more about the symptom-based algorithmic approaches contained in the clinical tool and apply it to their health promotion role.

### Trainer and mentor support

Development of program structures that facilitated peer support for HC trainers improved PALM PLUS program delivery and allowed for sustained engagement of motivated trainers, which was key to PALM PLUS program success. This motivation was sustained through active engagement and support to trainers by bringing them together for team trainings and quarterly meetings, and through supervision visits, and telephone and SMS contact. Trainers reported that they valued being linked to a supportive network of trainers and coordinators through these activities. There were no individual financial incentives offered to HC trainers at any time during the implementation of the PALM PLUS package.

Staff turnover of both the master trainer and HC trainers left gaps in training capacity until new trainers could be recruited and trained. For example, a second master trainer was identified out of the pool of HC trainers and had started the master training certification process in 2011, but had not completed it as of December 2011. Future iterations of the PALM PLUS approach should include plans to build extra training capacity and risk mitigation strategies for potential staff turnover early in the implementation planning process. Due to the staff turnover and large geographic distances across the district, seven cluster groups were created mid-way through the implementation process, to help facilitate face-to-face interactions between HC trainers at different sites and encourage peer-based support for HC trainers. This was also a way for ‘old’ trainers to mentor ‘new’ trainers through joint training, particularly in facilities without trainers or those with few staff, which provided a key support to small, remote HCs in particular. In addition to the cluster groups, quarterly trainer meetings were established to bring all the trainers from the district together and provided all HC trainers an opportunity to meet in person to share experiences, challenges, and opportunities, which was reported by trainers to strengthen inter-professional bonds.

### Health system support

During the implementation process, a procedure was established to enable PALM PLUS participants and HC trainers to receive CPD credit with the Malawi College of Nursing or the Malawi Medical Council, whereby PALM PLUS participants (nurses, medical assistants, and clinical officers) were eligible for one CPD credit per hour of educational outreach session and all HC trainers were eligible for two CPD credits per hour of facilitation of educational outreach sessions. The linkage of CPD credits reinforced the uptake and delivery of the educational outreach session as reported by PALM PLUS participants and HC trainers.

An in-service pilot scheme was initiated as a trial by establishing mechanisms and systems for the DHO to allocate district implementation plan funds directly to health facilities through PALM PLUS trainers. With the DHO, in-service guidelines were developed to assist trainers to develop plans and budgets, and account for costs incurred. Funds only covered training costs (i.e. transport refund for off-duty staff, airtime, supplies, and materials) and could not be paid to on-site trainers or trainees. Trainers received transport refunds to train at facilities other than their own and received a lunch allowance when intensive training sessions (four or more hours) were conducted. The budget for this in-service pilot scheme was small, approximately 300 Malawian Kwacha (less than one US Dollar) per person, per session. In spite of the small amounts, these refunds and allowances also strengthened uptake and delivery of the educational outreach sessions, as HCWs reported that they were motivated by this small token of support.

As the implementation of the PALM PLUS package evolved, it became necessary to reexamine the use of paper training logs. Major challenges to good record keeping for training were the large number of educational outreach sessions and staff turnover, especially for staff transferring between HCs, and there were concerns about unequal distribution of allocation of training opportunities for HC staff. Therefore, the TrainSMART system, an open-source, web-based training data collection system, was deployed to monitor educational outreach sessions for the PALM PLUS package and to record other trainings attended by PALM PLUS participants [9]. The TrainSMART system is managed by I-TECH and its use has been endorsed by the MoH.

## Discussion

In Malawi’s Health Sector Strategic Plan for 2011 to 2016, there is a call for innovative approaches to HCW training and development, including in-service training options [10]. The PALM PLUS package is a potential model of educational outreach, a form of in-service training, to support existing human resources in providing front-line integrated primary care in Malawi and other low and middle-income countries facing challenges with human resources for health.

The original PALSA approach, on which the PALM PLUS package was based, was one of the first attempts at combining educational outreach with guideline implementation in sub-Saharan Africa [11]. Results from the PALSA intervention showed an improvement in case detection for tuberculosis in the intervention group [6]. The next iteration of PALSA, called Practical Approach to Lung Health Plus HIV/AIDS in South Africa (PALSA PLUS), was shown to better integrate AIDS care within a nurse-led primary health care clinic [12]. PALSA PLUS then evolved into a more discretely task-shifting initiative, Streamlining Tasks and Roles to Expand Treatment and Care for HIV (STRETCH); this focused on expanding primary-care nurses’ roles to include prescribing antiretroviral treatment [13]. The PALSA, PALSA PLUS, and STRETCH approach is considered a model for successful task-shifting in South Africa, and has now been evolved to an integrated primary care guideline and training initiative called Primary Care 101, which is currently being evaluated in a pragmatic randomized controlled trial [14].

The development of PALM PLUS was the first time that the PALSA program and its other variations was applied outside of South Africa. The main difference between PALM PLUS and PALSA and associated programs was that PALM PLUS focused on supporting existing mid-level HCW with their existing roles and responsibilities in primary health care, as task-shifting for HIV/AIDS care (including antiretroviral treatment) was already being done by mid-level HCW in Malawi--through necessity due to the extreme lack of human resources for health in Malawi at the HC level.

There is a paucity of published literature around supporting mid-level HCW in low and middle income countries; rather there is clustering of evidence around educational outreach for physicians and task-shifting to community health care workers [15]. In many resource-limited settings, mid-level HCW are the backbone of primary health care and it is essential to continue to find sustainable methods for supporting the indispensable role of this HCW cadre in primary care integration.

## Conclusions

In addition to the development of a locally adapted clinical tool and educational outreach approach, the PALM PLUS package relies on peer-based support networks, adequate training capacity, linkages with continuing professional development accreditation, and very modest in-service training budgets for optimal implementation, where the goal of implementation is continuing professional development of mid-level HCW to support them in providing front-line integrated primary care. One of the major limiting factors in successful implementation of the PALM PLUS package was staff turnover and desire for training allowances. These lessons learned could be more broadly implemented in other training approaches that support HCWs in low and middle-income countries.

Future plans for the PALM PLUS package will include analysis and dissemination of the associated cluster-randomized trial, and dissemination of the study results will include knowledge translation with key stakeholders and policymakers in Malawi. Additionally, there is potential to expand the PALM PLUS package in Malawi to incorporate a wider range of primary care conditions for middle-level HCWs, to employ it in the family medicine undergraduate medical school curriculum, and to adapt it for use with other HCW cadres, such as health surveillance assistants.

As an example of South-South collaboration for knowledge translation, the PALSA and PALM PLUS approaches also have potential to address disparities in health in high income countries where access to high quality health services may be limited due to human resource constraints in remote or underserviced communities.

## Abbreviations

ART: Antiretroviral treatment; CPD: Continuing professional development; DHO: District health office; HC: Health centre; HCW: Health care worker; KTU: Knowledge translation unit; MoH: Ministry of Health; PALM PLUS: Practical Approach to Lung Health Plus HIV/AIDS in Malawi; PALSA: Practical Approach to Lung Health in South Africa; PALSA PLUS: Practical Approach to Lung Health Plus HIV/AIDS in South Africa; STRETCH: Streamlining Tasks and Roles to Expand Treatment and Care for HIV; TtTtT: Training the trainers to train

## Competing interests

The authors declare that they have no competing interests.

## Authors’ contributions

SS, MJS, EB, and MZ conceived the project. MJS and SS led the drafting for the protocol and grant applications. LF, FR, and ST led the guideline and training curriculum development process. SS, HB, MJS, EB, LM, and MZ participated in study design. DK, HB, and MJ helped design implementation, evaluation and content, and provided policy support. EM, HM, and EK assisted with program evaluation. SS led manuscript writing. All authors approved the final manuscript.

## Declarations

The publication costs associated with this article are funded by Foreign Affairs, Trade and Development Canada and the International Development Research Centre through the Global Health Research Initiative.

This article has been published as part of *BMC Health Services Research* Volume 14 Supplement 1, 2014: Uptake and impact of research for evidence-based practice: lessons from the Africa Health Systems Initiative's research component. The full contents of the supplement are available online at http://www.biomedcentral.com/bmchealthservres/supplements/14/S1
